# Path-programmable water droplet manipulations on an adhesion controlled superhydrophobic surface

**DOI:** 10.1038/srep12326

**Published:** 2015-07-23

**Authors:** Jungmok Seo, Seoung-Ki Lee, Jaehong Lee, Jung Seung Lee, Hyukho Kwon, Seung-Woo Cho, Jong-Hyun Ahn, Taeyoon Lee

**Affiliations:** 1 School of Electrical and Electronic Engineering, Yonsei University, 50 Yonsei-ro, Seodaemun-Gu, Seoul 120-749, Republic of Korea; 2 Department of Biotechnology, Yonsei University, 50 Yonsei-ro, Seodaemun-Gu, Seoul 120-749, Republic of Korea

## Abstract

Here, we developed a novel and facile method to control the local water adhesion force of a thin and stretchable superhydrophobic polydimethylsiloxane (PDMS) substrate with micro-pillar arrays that allows the individual manipulation of droplet motions including moving, merging and mixing. When a vacuum pressure was applied below the PDMS substrate, a local dimple structure was formed and the water adhesion force of structure was significantly changed owing to the dynamically varied pillar density. With the help of the lowered water adhesion force and the slope angle of the formed dimple structure, the motion of individual water droplets could be precisely controlled, which facilitated the creation of a droplet-based microfluidic platform capable of a programmable manipulation of droplets. We showed that the platform could be used in newer and emerging microfluidic operations such as surface-enhanced Raman spectroscopy with extremely high sensing capability (10^−15^ M) and *in vitro* small interfering RNA transfection with enhanced transfection efficiency of ~80%.

Functionally integrated microfluidic devices that allow various laboratory operations in multidisciplinary fields have attracted considerable attention due to their efficient and adjustable reactions with small amounts of samples^1^,^2^,^3^,^4^. Owing to the large spectrum of potential uses, advances in microfluidics over the past decade have achieved sophisticated functionality and in-depth strategies for fluid handling. Conventionally, microfluidic devices have been studied based on a continuous flow system composed of microchannels, which requires multiple components including pumps, valves and mechanical mixers. However, several inherent problems of the microchannel based system such as unintended flow patterns caused by particular boundary effects, limited flow velocities and the lack of reconfigurability, have become serious obstacle to sustainable development. Interest in the development of a droplet-based microfluidic system as an alternative platform has increased due to its benefits in terms of low sample consumption, rapid reactions and the capability of integration with other analytical techniques^5^,^6^,^7^,^8^. A fair number of approaches have been investigated for the individual control of droplets including electrowetting^6^,^8^,^9^, light-induced actuation^10^,^11^, magnetic fields^12^,^13^,^14^,^15^, electrostatic forces^16^,^17^, and dielectrophoresis^18^. However, the aforementioned techniques require the additional additives such as magnetizable particles in droplets and external electric/magnetic field sources; these may result in undesired reactions during the operation of the microfluidic devices, which frustrate the versatile applications.

Recently, bio-inspired superhydrophobic surfaces that exhibit unique surface wetting properties have been utilized in the droplet-based microfluidic systems, since liquids on the surfaces can exist in individually controllable droplet states^19^,^20^. Droplet manipulation on the superhydrophobic surface can be achieved by engineering of the surface’s chemical or structural properties, which are directly related to the surface wetting and adhesion. Malvadkar *et al.*^21^ described anisotropic textured superhydrophobic surfaces that facilitated the uni-directional transportation of water droplets owing to energy barrier principles, which caused wettability differences along the sliding direction. Li *et al.*^22^,^23^ and Seo *et al.*^24^,^25^ developed smart superhydrophobic surfaces with tunable surface wetting and adhesion properties by using organic and inorganic materials that are responsive to external stimuli such as temperature, light sources and gas. Wu *et al.* demonstrated a curvature-driven *in situ* switching of superhydrophobic state from the pinned to roll-down for water droplet transportation^26^. Nevertheless, droplet manipulation on the superhydrophobic surfaces has included only a limited set of simple fluidic operations, significantly hindering its practical usage in an open-channel, droplet-based microfluidic system.

Herein, we present a novel method to control the water droplet motions on a thin and stretchable superhydrophobic polydimethylsiloxane (PDMS) surface via the formation of a local dimple structure. We found that an as-fabricated flat superhydrophobic surface without deformation had uniform adhesive force to water (*F*_*adh*_) in all of the areas with a water contact angle (WCA) of 151 ± 3°. When the local dimple structure was formed by the applied vacuum pressure below the suspended superhydrophobic substrate, *F*_*adh*_ of the superhydrophobic surface could be locally changed by the generated positive/negative curvature of the dimple structure. Consequently, the changed *F*_*adh*_ and slope wall of the dimple structure enable the capturing and moving water droplets along the local dimple structure without any additives and external field sources, by the utilization of the gravitational force. Based on the real-time manipulation of the dimple structure, the *in situ* control of water droplet motions including droplet transportation, merging, mixing and analysis were accomplished. As a proof-of-concept experiment, we demonstrated a programmable platform capable of newer and emerging bio-chip applications such as highly sensitive surface-enhanced Raman spectroscopy (SERS) measurements up to 10^−15^ M of sensing capability and *in vitro* small interfering RNA (siRNA) transfection with enhanced transfection efficiency of ~80%, due to the increased homogeneous mixing of transfection complexes on the platform.

## Results

### Fabrication of a superhydrophobic substrate for a droplet-based microfluidic platform

**Figure 1:**
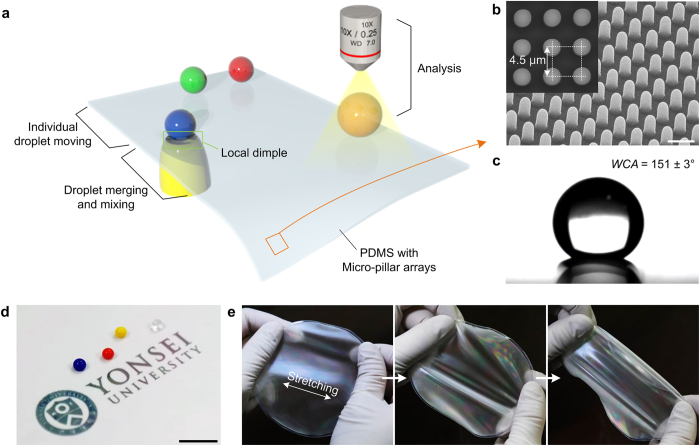
Superhydrophobic PDMS with micro-pillar arrays for manipulations of water droplet motion. (**a**) Schematic illustration of the microfluidic platform that used a local dimple structure to manipulate water droplet motions including moving, mixing and analysis on the suspended PDMS substrate with micro-pillar array. (**b**) SEM image of a regular micro-pillar arrays (2.5 μm radius, 4 μm height). Scale bar, 5 μm. (**c**,**d**) Photographs of water droplets on the surface of the PDMS substrate with micro-pillar arrays. Scale bar, 1 cm. (**e**) Photograph images showing the excellent stretchability of the PDMS substrate.

Figure 1a shows a schematic illustration of our open-channel, droplet-based microfluidic platform, which enables the individual control of droplet motions including moving, merging, mixing and analysis via the simple formation of a local dimple structure. PDMS was used as a substrate due to its excellent flexibility, superior chemical stability, and biocompatibility^27^. A wafer-scale, thin PDMS substrate with micro-pillar arrays was successfully fabricated through a single moulding step (Supplementary Fig. 1). Figure 1b illustrates a typical scanning electron microscope (SEM) image of the regular PDMS micro-pillar arrays with 4.5 μm periods. The micro-pillar arrays that were designed with round tips could effectively reduce the contact area between the substrate and the water droplets, which induced superhydrophobicity with an extremely large WCA of 151 ± 3° (Fig. 1c,d). Owing to the thin (275 μm thick) and flexible nature of the PDMS substrate, the fabricated substrate with micro-pillar array could provide excellent reversible stretchability without residual distortion (Fig. 1e).

### Formation of vacuum induced local dimple structure

**Figure 2:**
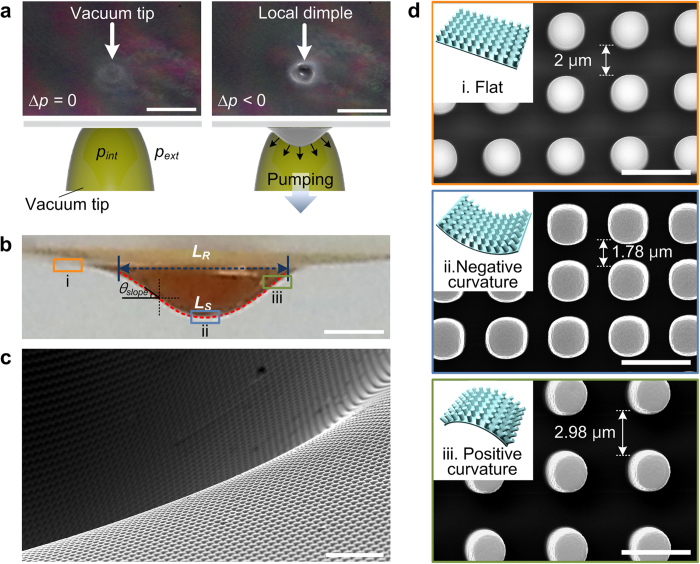
Vacuum induced local dimple formation on the PDMS substrate with micro-pillar arrays. (**a**) Photographic images and schematic illustrations of the local dimple formation on the substrate using a vacuum tip. Scale bar, 5 mm. (**b**) Cross-sectional photographic image of the negative replica of the dimple structure. PDMS substrate is uniformly stretched by the applied vacuum pressure. Scale bar, 1 mm. (**c**) Typical SEM image of the duplicated dimple structure. Scale bar, 30 μm. (**d**) SEM images of the micro-pillar arrays taken from the flat region (i), negative curvature region (ii) and positive curvature region (iii). Scale bar, 5 μm.

To manipulate individual water droplet motions on the superhydrophobic PDMS substrate, a millimeter-scale dimple structure was utilized that generated a local deformation of the substrate. Figure 2a includes photographic images and schematic illustrations of the formation of the local dimple structure on the PDMS micro-pillar arrays using a vacuum tip with a diameter of 2.85 mm, which directly contacted the underside of the PDMS substrate. Without the application of vacuum pressure, the pressure inside the vacuum tip (*p*_int_) was equal to the external atmospheric pressure (*p*_ext_) of 101 kPa. When the vacuum pressure was applied, a significant pressure difference, ∆*p* = *p*_int_ − *p*_ext_, was generated across the PDMS substrate. Due to the generated pressure difference, the PDMS substrate was stretched and deflected downward, forming the local dimple structure. In this work, ∆*p* was fixed at −81 kPa for the sake of convenient analysis. Considering the circular shape of the vacuum tip, it can be assumed that the pressure and corresponding strain stress were uniformly distributed across the entire surface of the dimpled PDMS substrate^28^. To characterize the structural changes of the vacuum-induced dimple structure, duplicated mould of the dimple structure were used (see Methods and Supplementary Fig. 2). Figure 2b,c show the optical photographic and SEM images of the negative replica’s cross-sectional image and duplicated dimple structure, respectively. The dimple structure caused the local stretching of the PDMS substrate as the substrate deflection formed a hemispherical shape. The degree of substrate stretching can be obtained by calculating the ratio of the relaxed (*L*_*R*_) to the stretched (*L*_*S*_) characteristic length of the dimple structure, as measured from the cross-sectional image of the negative replica. When the diameter of the vacuum tip was 2.85 mm, the measured substrate stretching (100 × (*L*_*S*_ − *L*_*R*_)/*L*_*R*_) was 20.7%. Due to the hemispherical shape of the dimple structure and the thickness of the PDMS substrate, negative and positive curvatures were generated at the bottom and border of the dimple structure, respectively. This local stretching of the substrate and the generation of the negative and positive curvatures enabled the dynamic changes in the distance between adjacent micro-pillars (*ε*_*dist*_), which is proved by the magnified SEM images (Fig. 2d) of the micro-pillar arrays taken from the flat region (Fig. 2di), the negative curvature region (Fig. 2dii) and the positive curvature region (Fig. 2diii). On the flat region (*ε*_*dist*_ = 0%), the distance between adjacent pillars was 2 μm. On the local dimple structure, *ε*_*dist*_ could be dynamically changed due to the combined effects of the substrate stretching and the positive/negative curvatures; the maximum value of *ε*_*dist*_ is 49% for the positive curvature region and the minimum value is −11% for the negative curvature region.

### Tuning the structural properties of local dimple structures

**Figure 3:**
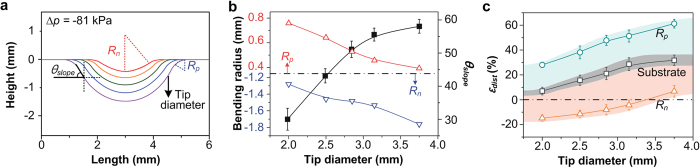
Geometric deformation of dimple as a function of vacuum tip diameter. (**a**) Cross-sectional profiles of local dimple versus position for five different diameters of vacuum tips. (**b**) Measured positive/negative bending radii and slope angles of dimple structures as a function of tip diameter. Red, blue and black lines indicate the positive bending radius, negative bending radius and slope angle, respectively. (**c**) The variation in *ε*_*dist*_ as a function of tip diameter.

To further identify the local deformation of the PDMS film with micro-pillar array, we controlled the bending radius of positive/negative curvature (*R*_*p*_/*R*_*n*_) and the slope of the dimple structure (*θ*_*slope*_), by varying the sizes of the vacuum tips. Figure 3a represents a series of cross-sectional profiles of the dimples as a function of tip diameter under the same pressure condition (∆*p* = −81 kPa). Due to the high mechanical strength and elasticity of PDMS, the substrate was stretched from ~7 to ~32%, when the diameters of the vacuum tips were increased from 2 to 3.75 mm, forming positive/negative curvatures with corresponding bending radii *R*_*p*_/*R*_*n*_. Figure 3b shows the results of the extracted values of the bending radii and slopes of the dimple structures from the cross-sectional profiles in Fig. 3a. *R*_*n*_ and *R*_*p*_ indicate the minimum radii at the center and border of the dimple, respectively, and *θ*_*slope*_ represents the steepest angle on the side of the dimple. The absolute value of *R*_*p*_ decreased from ~0.8 to ~0.4 mm as the diameter of the tip became larger, while the absolute value of *R*_*n*_ gradually increased from ~1.3 to ~1.8 mm as the tip diameter increased. The structural changes in the dimple structures that were dependent on tip size directly affect *ε*_*dist*_ of micro-pillars. To investigate *ε*_*dist*_ between the neighboring pillars depending upon *R*_*p*_/*R*_*n*_, we measured *ε*_*dist*_ of micro-pillars from the SEM images taken from positive and negate curvatures of the duplicated dimple structures (Supplementary Fig. 3). As shown in Fig. 3c, even though the substrate was deformed only between ~7 and ~32% by tensile stress caused by substrate stretching, the lateral distance between the neighboring pillars that we can adjust would be enlarged from −15 to 61% due to additionally generated deformations by a micro-pillar placed on the positive/negative curvature. For example, when the diameter of the tip is 2 mm, the distance between adjacent pillars at the positive curvature will be 28% broader than in the flat state, whereas the negative curvature has a 15% denser pillar interval. The grey, blue and orange colored region represents variations of substrate stretching, *ε*_*dist*_ at positive and negative curvature, respectively. The experimental results showed good agreement with the calculated *ε*_*dist*_, that considered the substrate stretching, *R*_*p*_, and *R*_*p*_. (Supplementary Fig. 4).

### Droplet manipulation by dynamic control of water adhesion force

**Figure 4:**
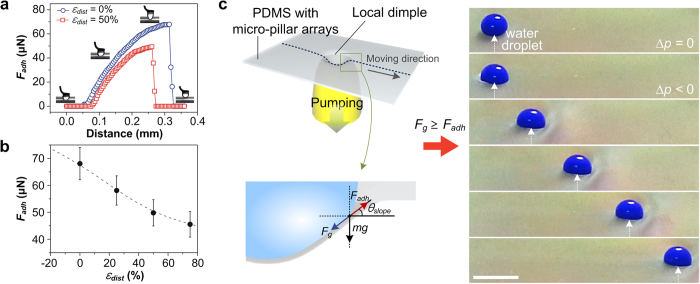
Dynamic water adhesion force changes for the manipulation of droplet motions on the PDMS substrate with micro-pillar arrays. (**a**) The force-distance curves for the PDMS substrate contacted with a water droplet. (**b**) Relationship between *F*_*adh*_ and *ε*_*dist*_ on the PDMS substrate. *F*_*adh*_ is decreased as *ε*_*dist*_ is increased. (**c**) Schematic illustration of the forces on the surface of a dimple structure that affect water droplet motions (left) and time-sequential photographic images of a moving water droplet on the PDMS substrate via the tunable dimple structure (right). Scale bar, 3 mm.

Since the changing effects of *ε*_*dist*_ has a sensitive correlation with the water adhesion force^26^, we investigate the dynamic *F*_*adh*_ changes on the dimple structure. Figure 4a depicts the force-distance curves for the PDMS substrate obtained with different values of *ε*_*dist*_. For these measurements, a water droplet suspended at a metal ring was attached and detached from the flat PDMS micro-pillar arrays to measure the force between the water droplet and the substrate. The measured force was gradually increased after contact and reached a maximum just before the contacted droplet was separated from the substrate. *F*_*adh*_ of the PDMS substrate with micro-pillar arrays was significantly decreased from 67 to 49 μN as *ε*_*dist*_ increased up to 50%. Figure 4b shows the measured *F*_*adh*_ as varying *ε*_*dist*_. It could be clearly observed that the *F*_*adh*_ of the PDMS micro-pillar arrays was gradually decreased as *ε*_*dist*_ increased. These *F*_*adh*_ changes according to *ε*_*dist*_ could be attributed to the variations in contact area between the micro-pillar arrays and the water droplet on the substrate. Theoretically, the wetting on the superhydrophobic PDMS micro-pillars without stretching is similar to the Cassie-Baxter wetting model, where air pockets exist between a droplet and a rough surface^29^; in this model, *F*_*adh*_ is proportional to the number of pillars in contact with the droplet^30^,^31^,^32^. Therefore, when the stretching-induced strain stress was applied to the PDMS substrate, the number of micro-pillars that were directly contacting the water droplet could be reduced, resulting in decrease of *F*_*adh*_.

Figure 4c is a schematic illustration of the forces on the surface of the dimple structure affecting the water droplet motions (left) and time-sequential photographic images of a blue-dyed, 10 μl moving water droplet on the PDMS micro-pillar arrays being moved via the controllable dimple structure (right). When the dimple structure was formed, the water droplet could be put into the structure and it seemed that the water droplet was spread along the dimple structure. Thus, the overall contact area of the water droplet with dimple structure was increased and the corresponding adhesion force between the water droplet and the surface was also increased. However, at the border of the dimple structure, the water droplet can be easily detached from the substrate due to the decreased local *F*_*adh*_ and generated sloped wall-induced gravitational force (*F*_*g*_ = *mg*sin *θ*_*slope*_). Here, *m* is the mass of the water droplet and *g* is the gravitational acceleration constant. When *F*_*g*_ became larger than the local *F*_*adh*_ at the boundary of dimple, the droplet detachment from the substrate continuously occurred during the horizontal moving of the dimple structure. Consequently, the water droplet could be moved along with the moving dimple structure (Supplementary Fig. 5). We observed that the controllable droplet volume could be determined by the diameter of vacuum tip (Supplementary Fig. 6). A larger vacuum tip could manipulate smaller water droplet, which can be attributed to the smaller *F*_*adh*_ and larger *θ*_*slope*_ of the generated dimple structure. When the volume of water droplets were larger than the minimum volume, the motions of the water droplets could be successfully manipulated even when the diameter of a water droplet was larger than the tip size due to the surface tension of the water droplet.

### Operation of the superhydrophobic, droplet-based microfluidic device

**Figure 5:**
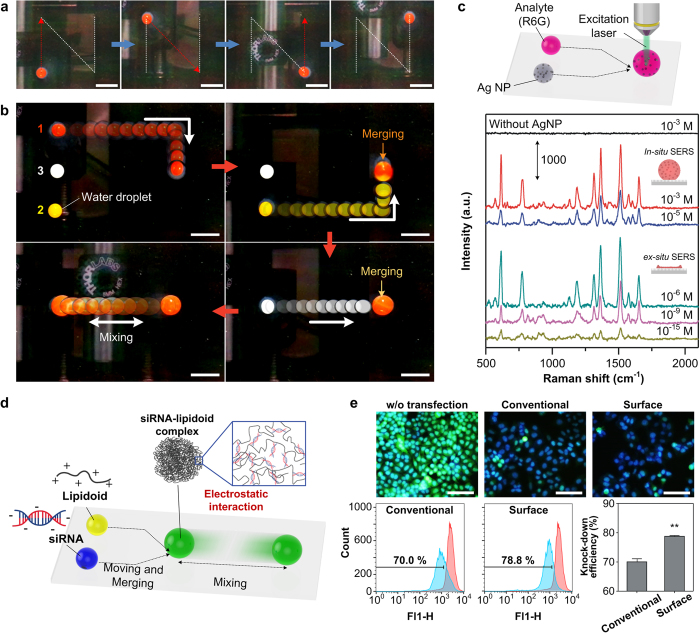
*In situ* manipulation of water droplet motions on the PDMS micro-pillar arrays for droplet-based microfluidic operations. (**a**) A 10 μl moving water droplet follows the trace of a character “N” shape. Scale bar, 5 mm. (**b**) Droplet operations including transportation, merging and mixing on the superhydrophobic PDMS substrate. Scale bar, 5 mm. (**c**) Scheme of the SERS measurement system (top) and typical *in situ*/*ex situ* SERS analysis spectra with different concentrations of analyte (R6G), obtained from a droplet mixture of R6G/Ag NP and evaporated R6G/Ag NP droplet, respectively (bottom). (**d**) Scheme of the siRNA-lipidoid complex formation for *in vitro* transfection. (**e**) Fluorescent images (top) and flow cytometry analyses of GFP-HeLa cells two days after transfection (bottom). Scale bar, 200 μm (n = 3, **p < 0.01, compared to the conventional group).

*In situ* manipulation of droplet motions including moving, merging and mixing was demonstrated on the superhydrophobic, droplet-based microfluidic platform. Since the motions of water droplets on the platform are controlled by the vacuum-induced dimple structure, the moving path can be freely designed without additional patterning process (Fig. 5a). These individually controllable droplet movements can be used for merging and mixing operations of droplet (Fig. 5b and Supplementary Movie 1). The capture and release of water droplets during the operations could be controlled by adjusting the applied pressure of the vacuum tip. When transported droplets were brought into proximity, they merged and mixed slightly as forming one large droplet. The complete mixing of the water droplet could be achieved by moving the water droplet back and forth on the superhydrophobic PDMS micro-pillar arrays. The mixing process could be attributed to the rolling effect on the dimple structure, which may generate internal hydrodynamic flows in the moving water droplets. As previously mentioned, the water droplet manipulation could be achieved by the slope angle and variations in *F*_*adh*_ of the dimple structure; therefore, a water droplet on the moving dimple structure could be continuously rolled along the slope wall of the dimple structure, following the trace of the dimple structure. Our method for the manipulation of droplet motions takes advantage of the fact that the pathway of the water droplet motion is freely designed and additional additives are not required to be added into the water droplets during the operations.

### *In situ* and *ex situ* SERS measurement

The simple and intuitive mechanism of our system enables an efficient reaction and analysis capability using microliter-sized analytes or reagents. The programmable bio-chip platform could be applied to SERS measurements, which enable the detection of a few molecules in a highly diluted solution^33^. Figure 5c shows the schematic of the droplet-based SERS measurement system and *in situ*/*ex situ* Raman spectra of Rhodamine 6G (R6G) with concentrations ranging from 10^−3^ to 10^−15^ M. For the *in situ* SERS measurement, individual water droplets containing the target molecules and Ag nanoparticles (Ag NPs) which exhibit a wide resonance spectrum wavelength range were merged at the detection spot using the dimple structure, and then signals were collected from the droplet mixture. As shown in Fig. 5c, several typical peaks for R6G can be identified such as the C−C−C ring in plane bending (612 cm^−1^), C−H out of plane bending (773 cm^−1^), aromatic C−H bending (1183 cm^−1^), C−O−C stretching (1312 cm^−1^) and C−C stretching (1363, 1511, 1575 and 1650 cm^−1^)^34^. We note here that the locations of observed peaks were identical to the previous work (Supplementary Fig. 7)^34^. In addition, droplet mixture could be removed without any residues remaining on the substrate, which enable the highly repeatable and reproducible *in situ* SERS measurements on the same substrate (Supplementary Fig. 8). However, *in situ* SERS measurements on the droplet-based microfluidic platform had a detection limit of 10^−5^ M due to freely diffusing Ag NPs and target molecules in the water droplets, which hindered the binding of the molecules to the surface of the Ag NPs^33^. To overcome the detection limit, the droplet mixture was evaporated on the surface over time until water was fully evaporated^35^. On a conventional hydrophilic SERS substrate, solution with target molecules could be spread out along the surface during the evaporation process and only few target molecules are located on the detection spot. In contrast, on the superhydrophobic surface, the contact area between the droplet and the surface can be minimized during the evaporation process due to the large contact angle^35^. As evaporation proceeded, the R6G/Ag NP solution became more and more concentrated and the target molecules and Ag NPs accumulated within an area of hundreds of square micrometres. After full evaporation, the target molecules were highly enriched on the Ag NPs detection spot, which permitted extremely high sensing capability even at femtomolar levels (10^−15^ M) for the *ex situ* SERS measurements. This detection limit is a hundred-fold lower than conventional SERS measurements on the flat substrate^34^. The obtained detection limit is poorer than the previously reported SERS measurements on superhydrophobic nanosensors^35^, due to the water adhesive property of the PDMS micro-pillar arrays. It is believed that the detection limit of our platform could be improved by using the water-repellent superhydrophobic surface since it enables the more enrichment of target molecules within smaller area.

### *In vitro* siRNA transfection

The superhydrophobic surface allowing for the intuitive movement of droplets was further tested by generating uniform complexes composed of gene and vector for intracellular gene transfer. For decades, a great effort has been made to develop non-viral gene delivery vectors that can replace viral vectors, which have inherent safety concerns including tumorigenicity, immunogenicity, and insertional mutation^36^,^37^,^38^,^39^,^40^. Most of the non-viral gene delivery vectors based on cationic polymers or lipids have relied on manual pipetting or vortexing to formulate transfection complexes via electrostatic charge interactions between anionic genetic materials and cationic delivery reagents^36^,^41^,^42^. However, the manual handling of the droplets often results in the inefficient formulation of transfection complexes and loss of the samples^43^. Thus, we hypothesized that the homogeneous mixing of the droplets of genes and delivery vectors by automated operation on the superhydrophobic surface may be able to minimize the loss of the materials and also generate uniform complexes with higher transfection efficiency by increasing the chance of interaction between anionic and cationic materials. To test the potential application of our surface in the efficient formulation of transfection complexes, the surface was used to induce the formation of the complexes with lipidoid, a potent lipid-like material for gene delivery, and siRNA. Lipidoid has been identified as a highly effective siRNA delivery vector with a higher transfection efficiency and lower cytotoxicity than currently available transfection reagents^36^. Lipidoid can mediate siRNA transfer into various types of cells and tissues, and thus it has shown great potential for therapeutic applications in diverse diseases^44^,^45^,^46^,^47^,^48^.

The transfection efficiency of the lipidoid-siRNA complexes generated on our surface was compared with that of the complexes prepared by the conventional mixing method using manual pipetting. The complexes composed of lipidoid (ND98) and green fluorescent protein-siRNA (siGFP) for the transfection were prepared on the PDMS micro-pillar arrays by simply merging and mixing the solutions of lipidoid and siRNA via microfluidic operations (Fig. 5d). Two days after the transfection into GFP-HeLa cells, ~70% silencing of GFP expression (70.0 ± 1.9%) was observed in the cells transfected with the complexes prepared by the conventional mixing method (Fig. 5e). Interestingly, the formulation of the complexes on our surface further increased GFP silencing up to ~80% (78.8 ± 0.5%) (Fig. 5e). The automated precise handling of the droplets of lipidoid and siRNA solutions on our surface may induce the efficient formation of more homogeneous complexes, ultimately leading to the enhancement of siRNA transfection efficiency and GFP silencing. Considering the high intercellular gene transfer efficiency of GFP-HeLa cell^36^, ~10% of improvement in the transfection is meaningful and it might be further increase in the cells usually exhibiting low transfection efficiency such as stem cells or primary cells. Given that the superhydrophobic PDMS micro-pillar array enables the formation of homogeneous complexes with enhanced transfection capability and minimal loss of the materials, and is also compatible with an intuitive automated operation, this system would be useful as a high-throughput platform to screen the material candidates for effective gene delivery and produce genetic therapeutics for disease treatment.

## Discussion

Herein, we have developed and presented a novel way to manipulate droplets by dynamically controlling of the adhesion through the geometric deformation of the PDMS micro-pillar arrays. Due to the low water adhesion force and high stretchability of the superhydrophobic PDMS substrate with micro-pillar arrays, the structural modulation of the micro-pillars via local dimple structure could be amplified. The detailed distribution of pillar arrays according to the location of a local dimple was clarified by visual observation using SEM as well as by numerical calculations. Experimental and theoretical results revealed that the density difference of the pillar was more than 50% between the edge and center of the dimple; this difference can be utilized to manipulate the droplet to the desired position without any loss of weight. The actual measurement results show that the minimum adjustable capacity of the droplet was ~7 μl, which is a sufficient amount to be applied to programmable bio-chip devices. Moreover, the local deformation-based non-contact control method provides not only pure droplet manipulation without any additives, but also degrees of freedom on the surface, since additional path, patterns or additives are not necessary. These advantages facilitate the open-channel microfluidic operations such as ultra-sensitive molecular detection and siRNA transfection, which are not easy to achieve with conventional droplet-based, open-channel microfluidics. We foresee that our deformation-driven droplet manipulation on the superhydrophobic surface will have a significant impact on the evolution of next generation microfluidic systems for chemical and biological applications due to their advantages, including simple and clean manipulation without contact, multiple-degrees of freedom and good repeatability.

## Methods

### Fabrication of superhydrophobic PDMS micro-pillar arrays

The 4-inch wafer-scale Si mould with micro-holes (2.5 μm radius, 4 μm height) was fabricated by conventional photolithography and subsequent reactive ion etching. The surface of the Si mould was modified with a hydrophobic self-assembled monolayer (dodecyltrichlorosilane: DTS, Aldrich) by immersing the mould in a 3 mM solution of DTS dissolved in toluene for 30 minutes at room temperature. Then, the DTS-coated Si mould was rinsed with ethanol and baked at 130 °C for 1 h to obtain a dense DTS layer. Next, 5 g of polydimethylsiloxane (PDMS, Sylgard 184, Dow Corning), mixed with a curing agent at a volume ratio of 10:1, was spin-coated onto the mould (500 rpm, 10 seconds). To ensure the fine replication of the micro-pillar structures, the remaining air bubbles between the PDMS and the Si mould were removed in a vacuum chamber. The PDMS was cured at 70 °C for 2 h and carefully peeled off. The obtained PDMS micro-pillar arrays showed superhydrophobicity without further surface modification processes.

### Manipulation of droplet motions on PDMS micro-pillar arrays

The obtained PDMS substrate with micro-pillar arrays was suspended on a sample holder with an 85 mm-diameter central hole, without sagging of the substrate. The central hole, the actual area for the water manipulation, is smaller than the PDMS substrate because the adhesion area between the substrate and the mould is required to mount the substrate on the sample holder. A vacuum tip, which created a local dimple on the PDMS substrate, was placed beneath the substrate for the manipulation of droplet motions. The location of the vacuum tip was precisely controlled by a programmed translational stage.

### Fabrication of the negative replica and duplicated PDMS micro-pillar arrays

Firstly, a negative replica of the dimple structure, made of ultraviolet-curable photoresist (SU-8, Microchem), was obtained by ultraviolet light exposure for 30 min. Then, the negative replica was detached from the substrate and PDMS mixed with curing agent was poured onto the negative replica to fabricate the duplicated mould of the dimple structure.

### SERS measurements

Rhodamine 6G (R6G, Aldrich) was used as a probing molecule for the SERS measurements in solution of various concentrations (10^−3^, 10^−6^, 10^−9^, 10^−12^ and 10^−15^ M). An aqueous nanoparticle suspension containing Ag NPs ~80 nm in diameter was synthesized using the polyol process^49^. Then, 5 μl of the Ag NP suspension and R6G solution were dropped onto the PDMS substrate and each droplet was moved to the detection spot for SERS measurement using the droplet motion manipulating system. A focused He-Ne laser (633 nm, 2 mW) was used as the Raman excitation light source. The signals from each sample were collected for 1 s using a 10 × microscope objective (Olympus) and analyzed using a Raman spectrometer (Horiba-Jobin-Yvon, LabRam HR).

### *In vitro* siRNA transfection using lipidoid-siRNA complex formulation

GFP-expressing HeLa (GFP-HeLa) cells were cultured in Dulbecco’s Modified Eagle’s Medium (DMEM, Gibco BRL) supplemented with 10% (v/v) fetal bovine serum (FBS, Gibco BRL), penicillin (100 U/mL), and streptomycin (100 mg/mL) in humidified air with 5% CO_2_ at 37 °C. Lipidoid (ND98), a potent siRNA transfection reagent, was synthesized as previously described^36^,^48^. The lipidoid-siRNA complexes prepared by the conventional mixing method with pipetting^36^ were used for control transfection. To prepare the lipidoid-siRNA complexes on the PDMS micro-pillar arrays, ND98 lipidoid and GFP-siRNA (siGFP) were dissolved in the same volume of 25 mM sodium acetate (NaOAc) buffer solution (Sigma-Aldrich, pH 5.2). The droplets of each solution were placed apart on the PDMS micro-pillar arrays and then merged using the microfluidic operations. Subsequently, the merged droplets were further mixed by moving them side to side within a range of 30 mm for 5 min to induce complex formation. The mixed droplets were transferred into a tube right after the mixing and further incubated at room temperature for 15 min. The ratio of siGFP to ND98 in the formed complexes was 5:1 (w/w). The cells (2.5 × 10^4^ cells/cm^2^) were then transfected with the complexes (0.2 μg siGFP/cm^2^). To evaluate the GFP silencing two days after the siRNA transfection, GFP expression in the transfected cells was observed using a fluorescent microscope (IX71, Olympus). The fraction of GFP-positive cells was quantified by flow cytometry analysis. For the flow cytometry analysis, the cells were collected by trypsin treatment, washed with 1 × phosphate buffered saline (PBS, Sigma-Aldrich), resuspended in 2% (v/v) FBS (in PBS), and analyzed by a FACSCalibur flow cytometer (BD Biosciences, San Jose, CA, USA) (n = 3).

### Characterization

The surface morphologies of the PDMS micro-pillar arrays and duplicated PDMS structures were characterized using a field emission scanning electron microscope (JSM-6360, JEOL). Static WCA measured using a contact angle measurement system equipped with a dynamic image capture camera (Phoenix 300, SEO Co., Ltd.). To characterize structural properties of the dimple structure, cross-sectional images of negative replicas were obtained using the contact angle measurement system and values of the substrate stretching, *R*_*p*_, *R*_*n*_, and slope angles were measured by Image J software. *ε*_*dist*_ values were measured from the SEM images taken from the duplicated PDMS structures and compared with the calculation that considered the substrate stretching, *R*_*p*_, and *R*_*n*_. The water adhesion force of the PDMS with micro-pillar structures was measured by a home-made micro-electromechanical balance system. A 10-μl water droplet suspended from a hydrophobic metal ring was moved toward and retracted from the sample at a speed of 0.01 mm s^−1^. After the droplet was contacted to the substrate, it was dragged back from the substrate. The value of the measured force reached a maximum just before the contacted droplet separated from the substrate. To measure water adhesion force under stretched condition, micro-pillar arrayed PDMS substrates with different *ε*_*dist*_ (0, 25, 50, 75%) were fabricated using *ε*_*dist*_ tuned Si moulds.

## Additional Information

**How to cite this article**: Seo, J. *et al.* Path-programmable water droplet manipulations on an adhesion controlled superhydrophobic surface. *Sci. Rep.* **5**, 12326; doi: 10.1038/srep12326 (2015).

## Supplementary Material

Supplementary Video

Supplementary Information

## References

[b1] Daw R. & Finkelstein J. Lab on a chip. Nature 442, 367–367 (2006).

[b2] deMello A. J. Control and detection of chemical reactions in microfluidic systems. Nature 442, 394–402 (2006). 16871207 10.1038/nature05062

[b3] El-Ali J., Sorger P. K. & Jensen K. F. Cells on chips. Nature 442, 403–411 (2006). 16871208 10.1038/nature05063

[b4] Yager P. *et al.* Microfluidic diagnostic technologies for global public health. Nature 442, 412–418 (2006). 16871209 10.1038/nature05064

[b5] Choi K., Ng A. H. C., Fobel R. & Wheeler A. R. Digital Microfluidics. Annu. Rev. Anal. Chem. 5, 413–440 (2012). 10.1146/annurev-anchem-062011-143028 22524226

[b6] Abdelgawad M. & Wheeler A. R. The Digital Revolution: A New Paradigm for Microfluidics. Adv. Mat. 21, 920–925 (2009).

[b7] Fair R. B. Digital microfluidics: is a true lab-on-a-chip possible? Microfluid Nanofluid 3, 245–281 (2007).

[b8] Pollack M. G., Pamula V. K., Srinivasan V. & Eckhardt A. E. Applications of electrowetting-based digital microfluidics in clinical diagnostics. Expert Rev. Mol. Diagn. 11, 393–407 (2011). 21545257 10.1586/erm.11.22

[b9] Jebrail M. J. & Wheeler A. R. Let’s get digital: digitizing chemical biology with microfluidics. Curr. Opin. Chem. Biol. 14, 574–581 (2010). 20674472 10.1016/j.cbpa.2010.06.187

[b10] Chiou P. Y., Ohta A. T. & Wu M. C. Massively parallel manipulation of single cells and microparticles using optical images. Nature 436, 370–372 (2005). 16034413 10.1038/nature03831

[b11] Baigl D. Photo-actuation of liquids for light-driven microfluidics: state of the art and perspectives. Lab Chip 12, 3637–3653 (2012). 22864577 10.1039/c2lc40596b

[b12] Hong X., Gao X. & Jiang L. Application of superhydrophobic surface with high adhesive force in no lost transport of superparamagnetic microdroplet. J. Am. Chem. Soc. 129, 1478–1479 (2007). 17243677 10.1021/ja065537c

[b13] Khalil K. S., Mahmoudi S. R., Abu-dheir N. & Varanasi K. K. Active surfaces: ferrofluid-impregnated surfaces for active manipulation of droplets. Appl. Phys. Lett. 105, 041604 (2014).

[b14] Timonen J. V. I., Latikka M., Ikkala O. & Ras R. H. A. Free-decay and resonant methods for investigating the fundamental limit of superhydrophobicity. Nat. Commun. 4, 2398 (2013). 24025991 10.1038/ncomms3398

[b15] Zhang Y. & Wang T.-H. Full-range magnetic manipulation of droplets via surface energy traps enables complex bioassays. Adv. Mat. 25, 2903–2908 (2013). 10.1002/adma.201300383 PMC3964134 23529938

[b16] Zhou H. & Yao S. Electrostatic charging and control of droplets in microfluidic devices. Lab Chip 13, 962–969 (2013). 23338121 10.1039/c2lc41060e

[b17] Masahide G. & Masao W. Self-propulsion of a water droplet in an electric field. *J. Phys. D*: *Appl*. Phys. 38, 2417–2423 (2005).

[b18] Fan S.-K., Hsieh T.-H. & Lin D.-Y. General digital microfluidic platform manipulating dielectric and conductive droplets by dielectrophoresis and electrowetting. Lab Chip 9, 1236–1242 (2009). 19370242 10.1039/b816535a

[b19] Sun T., Feng L., Gao X. & Jiang L. Bioinspired surfaces with special wettability. Acc. Chem. Res. 38, 644–652 (2005). 16104687 10.1021/ar040224c

[b20] Sun T. & Qing G. Biomimetic smart interface materials for biological applications. Adv. Mat. 23, H57–77 (2011). 10.1002/adma.201004326 21433103

[b21] Malvadkar N. A., Hancock M. J., Sekeroglu K., Dressick W. J. & Demirel M. C. An engineered anisotropic nanofilm with unidirectional wetting properties. Nat. Mater. 9, 1023–1028 (2010). 20935657 10.1038/nmat2864

[b22] Li C. *et al.* Reversible switching of water-droplet mobility on a superhydrophobic surface based on a phase transition of a side-chain liquid-crystal polymer. Adv. Mat. 21, 4254–4258 (2009).

[b23] Li C. *et al.* *In situ* fully light‐driven switching of superhydrophobic adhesion. Adv. Func. Mat. 22, 760–763 (2012).

[b24] Seo J. *et al.* Gas‐driven ultrafast reversible switching of super‐hydrophobic adhesion on palladium‐coated silicon nanowires. Adv. Mat. 25, 4139–4144 (2013). 10.1002/adma.201300979 23733597

[b25] Seo J. *et al.* Switchable water-adhesive, superhydrophobic palladium-layered silicon nanowires potentiate the angiogenic efficacy of human stem cell spheroids. Adv. Mat. 26, 7043–7050 (2014). 10.1002/adma.201402273 25183387

[b26] Wu D. *et al.* Curvature-driven reversible *in situ* switching between pinned and roll-down superhydrophobic states for water droplet transportation. Adv. Mat. 23, 545–549 (2011). 10.1002/adma.201001688 21254261

[b27] Lee S. A., Chung S. E., Park W., Lee S. H. & Kwon S. Three-dimensional fabrication of heterogeneous microstructures using soft membrane deformation and optofluidic maskless lithography. Lab Chip 9, 1670–1675 (2009). 19495448 10.1039/b819999j

[b28] Yoon S.-H., Reyes-Ortiz V., Kwang-Ho K., Young Ho S. & Mofrad M. R. K. Analysis of circular PDMS microballoons with ultralarge deflection for MEMS design. J. Microelectromech. Syst. 19, 854–864 (2010).

[b29] Cassie A. B. D. & Baxter S. Wettability of porous surfaces. Trans. Faraday. Soc. 40, 546–551 (1944).

[b30] Wong T. S. & Ho C. M. Dependence of macroscopic wetting on nanoscopic surface textures. Langmuir 25, 12851–12854 (2009). 19842620 10.1021/la902430w PMC2783847

[b31] Extrand C. W. Model for contact angles and hysteresis on rough and ultraphobic surfaces. Langmuir 18, 7991–7999 (2002).

[b32] Dorrer C. & Rühe J. Contact line shape on ultrahydrophobic post surfaces. Langmuir 23, 3179–3183 (2007). 17269802 10.1021/la062596v

[b33] Ko H., Singamaneni S. & Tsukruk V. V. Nanostructured surfaces and assemblies as SERS media. Small 4, 1576–1599 (2008). 18844309 10.1002/smll.200800337

[b34] Lee J. *et al.* Capillary force-induced glue-free printing of Ag nanoparticle arrays for highly sensitive SERS substrates. ACS Appl. Mater. Interfaces. 6, 9053–9060 (2014). 24824186 10.1021/am5000382

[b35] De Angelis F. *et al.* Breaking the diffusion limit with super-hydrophobic delivery of molecules to plasmonic nanofocusing SERS structures. Nat. Photon. 5, 682–687 (2011).

[b36] Akinc A. *et al.* A combinatorial library of lipid-like materials for delivery of RNAi therapeutics. Nat. Biotechnol. 26, 561–569 (2008). 18438401 10.1038/nbt1402 PMC3014085

[b37] Soutschek J. *et al.* Therapeutic silencing of an endogenous gene by systemic administration of modified siRNAs. Nature 432, 173–178 (2004). 15538359 10.1038/nature03121

[b38] McNamara J. O. *et al.* Cell type–specific delivery of siRNAs with aptamer-siRNA chimeras. Nat. Biotechnol. 24, 1005–1015 (2006). 16823371 10.1038/nbt1223

[b39] Green J. J., Langer R. & Anderson D. G. A combinatorial polymer library approach yields insight into nonviral gene delivery. Acc. Chem. Res. 41, 749–759 (2008). 18507402 10.1021/ar7002336 PMC3490629

[b40] Guo X. & Huang L. Recent advances in nonviral vectors for gene delivery. Acc. Chem. Res. 45, 971–979 (2011). 21870813 10.1021/ar200151m PMC3240701

[b41] Lynn D. M. & Langer R. Degradable poly (β-amino esters): synthesis, characterization, and self-assembly with plasmid DNA. J. Am. Chem. Soc. 122, 10761–10768 (2000).

[b42] Park T. G., Jeong J. H. & Kim S. W. Current status of polymeric gene delivery systems. Adv. Drug. Deliver. Rev. 58, 467–486 (2006). 10.1016/j.addr.2006.03.007 16781003

[b43] De Smedt S. C., Demeester J. & Hennink W. E. Cationic polymer based gene delivery systems. Pharm. Res. 17, 113–126 (2000). 10751024 10.1023/a:1007548826495

[b44] Frank-Kamenetsky M. *et al.* Therapeutic RNAi targeting PCSK9 acutely lowers plasma cholesterol in rodents and LDL cholesterol in nonhuman primates. *Proc. Natl Acad*. Sci. USA 105, 11915–11920 (2008). 10.1073/pnas.0805434105 PMC2575310 18695239

[b45] Whitehead K. A. *et al.* Degradable lipid nanoparticles with predictable *in vivo* siRNA delivery activity. Nat. Commun. 5, 4277 (2014). 24969323 10.1038/ncomms5277 PMC4111939

[b46] Love K. T. *et al.* Lipid-like materials for low-dose, *in vivo* gene silencing. *Proc. Natl Acad*. Sci. USA 107, 1864–1869 (2010). 10.1073/pnas.0910603106 PMC2804742 20080679

[b47] Nguyen D. N. *et al.* Lipid-derived nanoparticles for immunostimulatory RNA adjuvant delivery. *Proc. Natl Acad*. Sci. USA 109, E797–E803 (2012). 10.1073/pnas.1121423109 PMC3325698 22421433

[b48] Cho S. W. *et al.* Lipid‐Like Nanoparticles for Small Interfering RNA Delivery to Endothelial Cells. Adv. Func. Mat. 19, 3112–3118 (2009). 10.1002/adfm.200900519 PMC3564673 23393492

[b49] Korte K. E., Skrabalak S. E. & Xia Y. Rapid synthesis of silver nanowires through a CuCl- or CuCl_2_-mediated polyol process. J. Mater. Chem. 18, 437–441 (2008).

