# Supplementary material for: Path-programmable water droplet manipulations on an adhesion controlled superhydrophobic surface
Source: Sci Rep. 2015 Jul 23;5:12326. doi: 10.1038/srep12326 (PMC4511949; doi:10.1038/srep12326)
Supplement: Supplementary Information [file srep12326-s2.pdf]

Supplementary Information

Path-programmable water droplet manipulations on an adhesion controlled superhydrophobic surface

Jungmok Seo[1,*], Seoung-Ki Lee[1,*], Jaehong Lee[1], Jung Seung Lee[2], Hyukho Kwon[1], Seung-Woo Cho[2], Jong-Hyun Ahn[1] and Taeyoon Lee[1]

[1]School of Electrical and Electronic Engineering, Yonsei University, 50 Yonsei-ro, Seodaemun-Gu, Seoul 120-749, Republic of Korea

[2]Department of Biotechnology, Yonsei University, 50 Yonsei-ro, Seodaemun-Gu, Seoul 120-749, Republic of Korea

* These authors contributed equally to this work.

Correspondence and requests for materials should be addressed to J.-H.A. (ahnj@yonsei.ac.kr) or T.L (taeyoon.lee@yonsei.ac.kr)

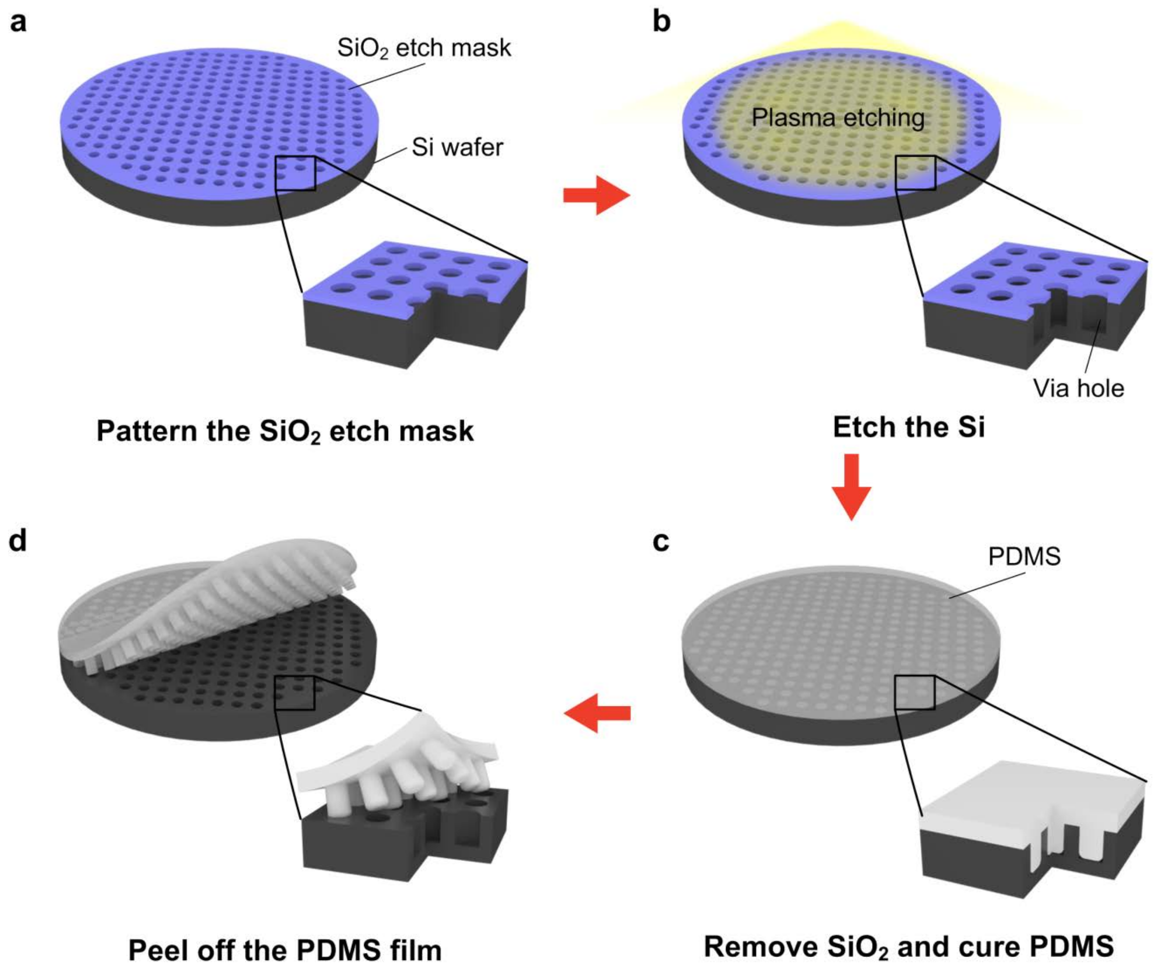

Supplementary Figure 1. Schematic illustration of the fabrication process of PDMS micropillar arrays. (**a**) SiO_2 etch mask formation. (**b**) Forming the via hole by etching of the Si wafer. (**c**) Removing SiO_2 etch mask and curing PDMS. (**d**) Peeling off the PDMS film with regular micropillar arrays.

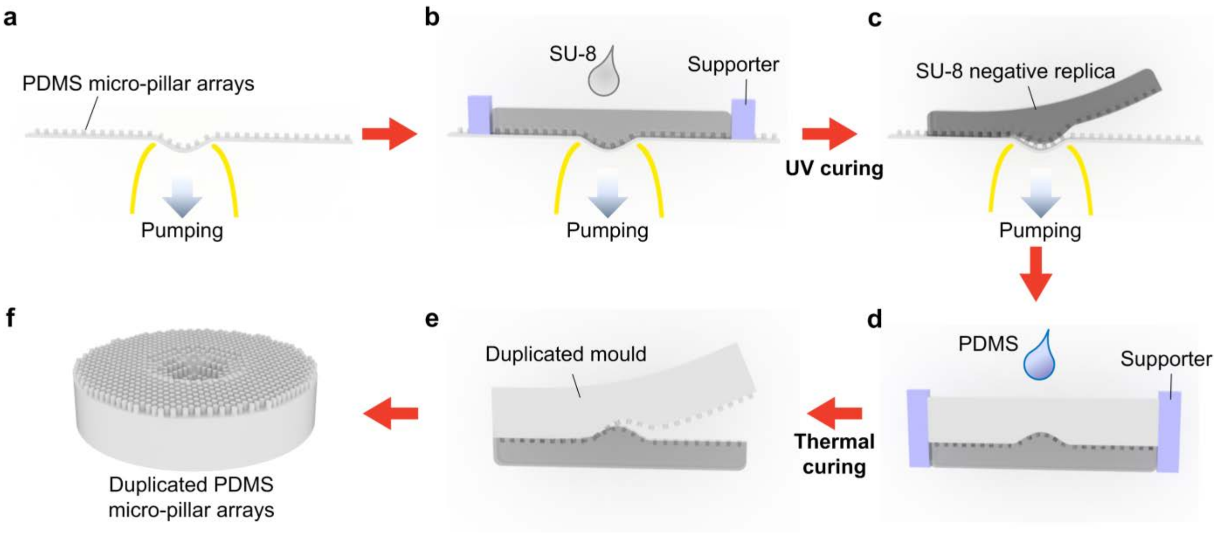

Supplementary Figure 2. Schematic of the fabrication process for negative replica and duplicated PDMS micropillar arrays. (**a**) Vacuum induced local dimple formation. (**b**) Pour SU-8 on the formed local dimple. (**c**) Peel off the SU-8 mould after UV curing. (**d**) Pour PDMS on the SU-8 negative replica. (**e**,**f**) Peel off the duplicated PDMS after thermal curing of PDMS.

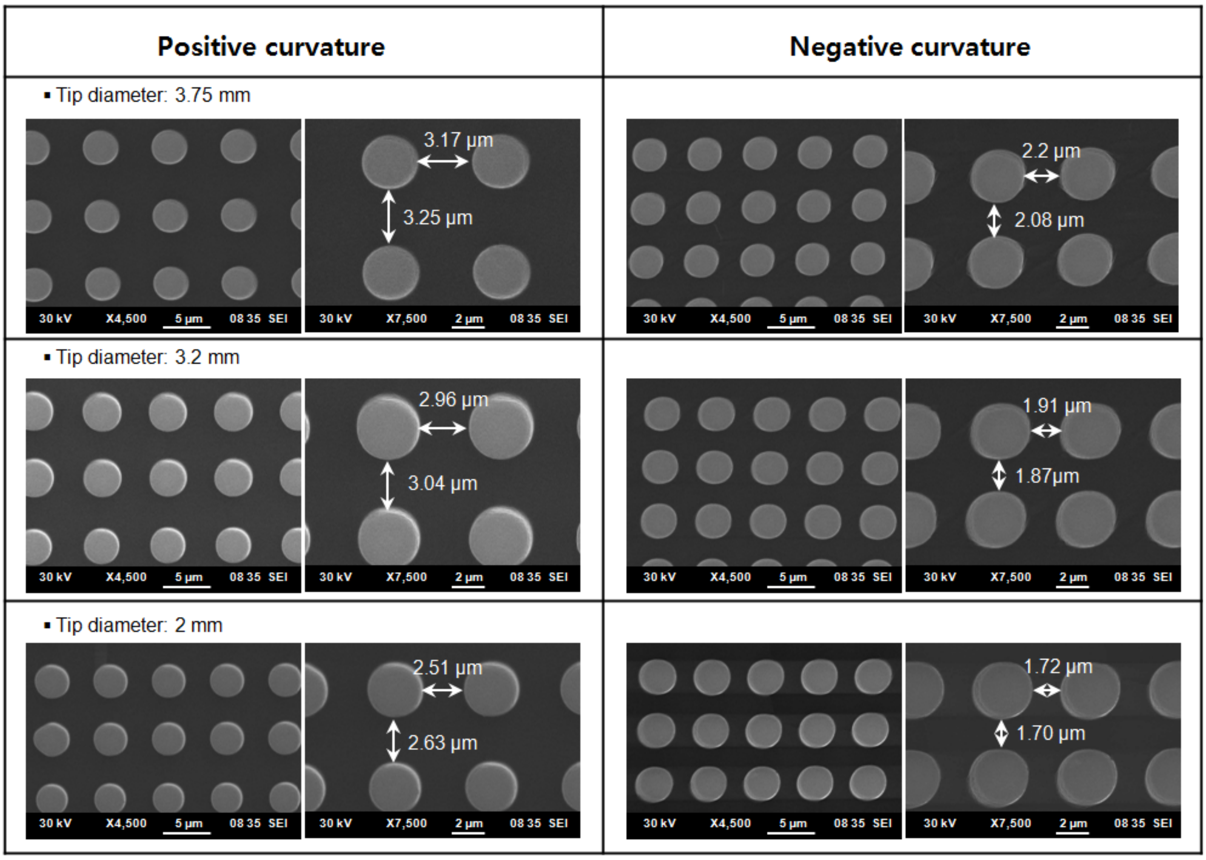

Supplementary Figure 3. Top view SEM image of the PDMS micropillar arrays at the positive and negative curvature of dimple structure as varying the tip diameter.

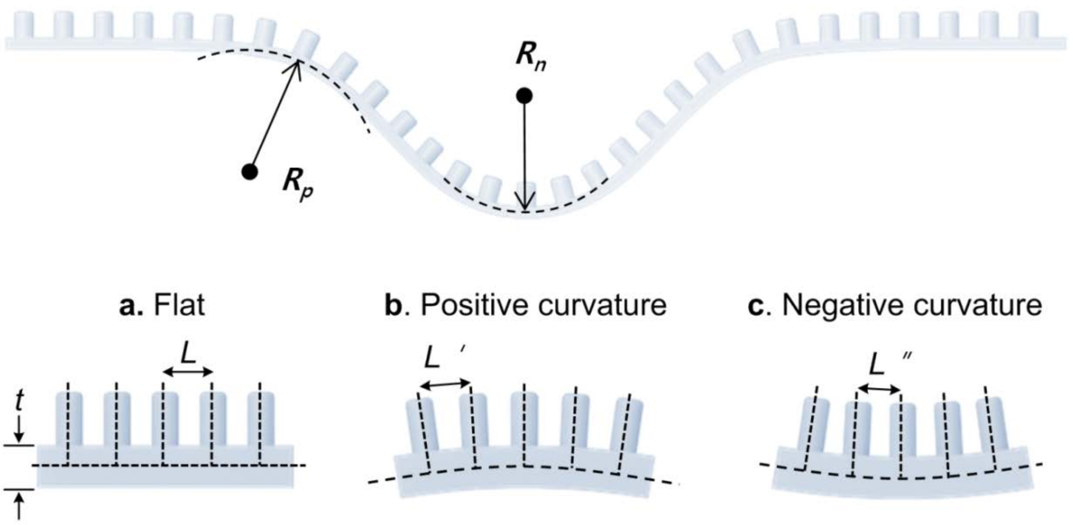

Supplementary Figure 4. Schematic cross sectional geometry of the PDMS with micro-pillar arrays for (**a**) flat, (**b**) positive and (**c**) negative bending state. The lateral distance (L) between the tops of neighboring pillars will either increase (L') or decrease (L''), depending upon whether the curvature is negative or positive. The variation of lateral distance of pillar when substrate is deformed by dimple can be calculated using the following equation:

$$\frac{\frac{t^2}{4}+h^2+Rt+2hR+ht}{R^2} \tag{1}$$

where t is the thickness of PDMS substrate, h is the height of pillar, and R is the bending radius of substrate.

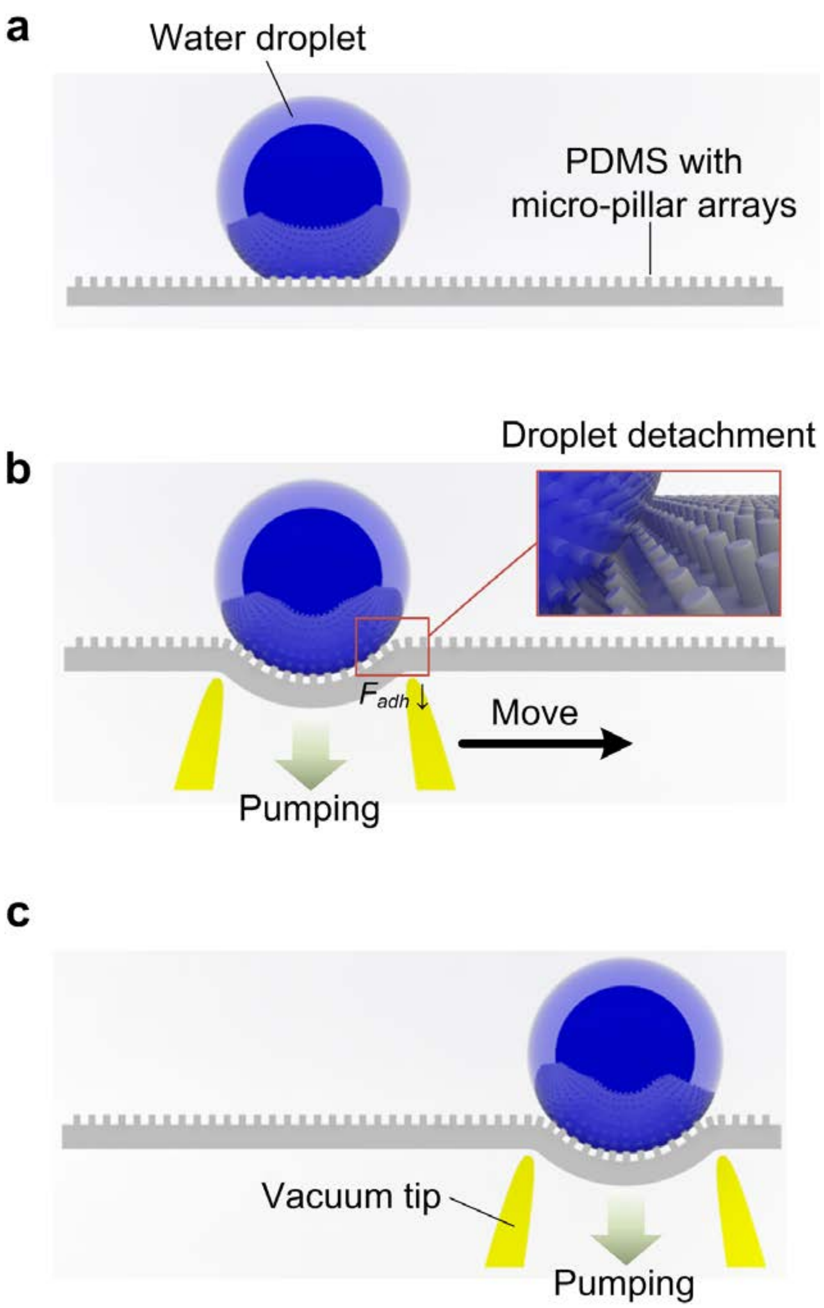

Supplementary Figure 5. Schematic of the manipulation of a water droplet using the vacuum-induced dimple structure. (**a**) A water droplet on the micro-pillar arrays. (**b**) Dimple structure formation and decreasement of F_{adh} at the border of dimple structure. When the vacuum tip moves horizontally, the contacted surface between the water droplet and the pillar arrays at the border can be detached. (**c**) Manipulation of water droplet using the vacuum tip.

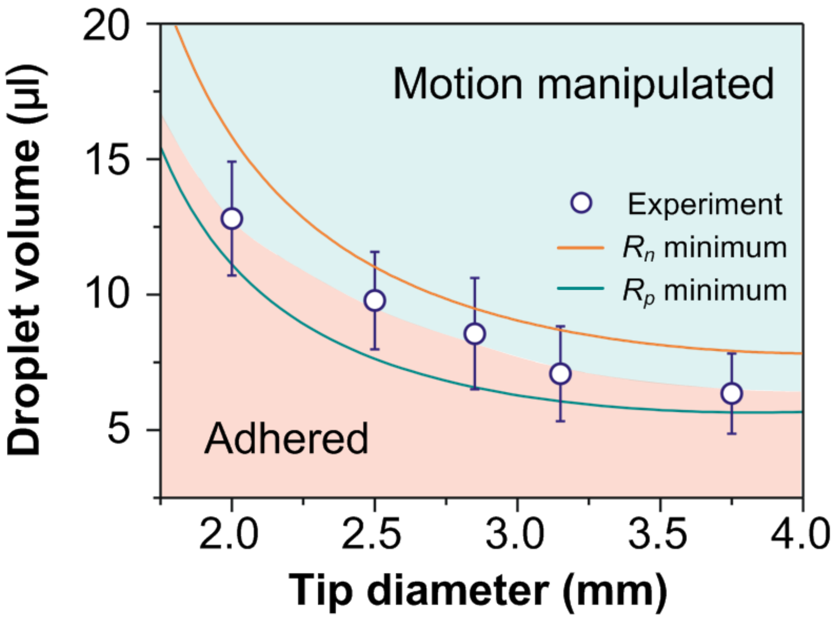

Supplementary Figure 6. Adjustable water manipulation diagram as a function of tip diameter. Here, R_n minimum and R_p minimum indicate the non-linear fitting curve of the minimum manipulatable droplet volume at positive (R_p) and negative curvature (R_n) of each tip, respectively. Theoretically, F_{adh} on the dimple structure has its maximum and minimum value at R_n and R_p, respectively. Therefore, by considering the slope angle and the range of F_{adh} on the dimple structure, the minimum manipulatable droplet volume can be ranged between R_n minimum and R_p minimum. The values of manipulatable droplet volume were calculated by using the measured slope angle (Figure 3b), positive and negative curvature (Figure 3b), equation of Supplementary Figure 4, and corresponding F_{adh} (Figure 4b).

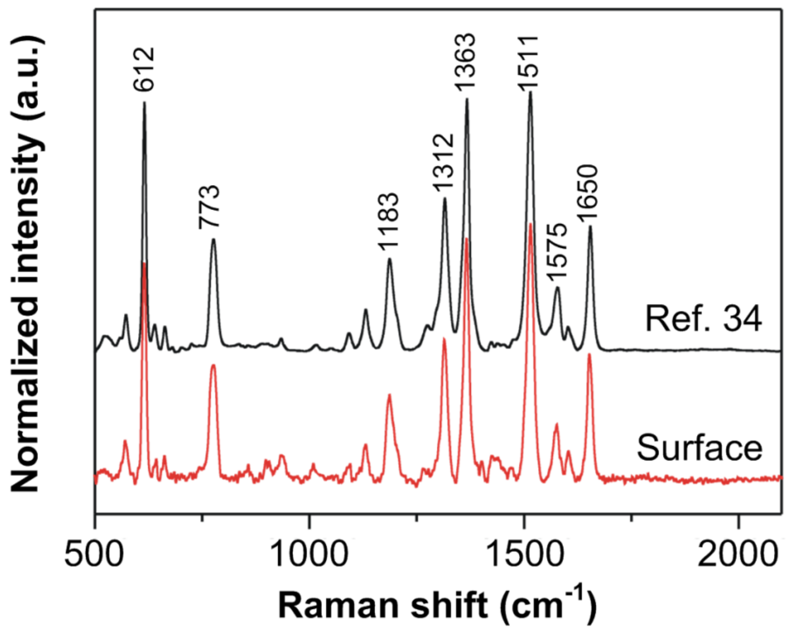

Supplementary Figure 7. Typical SERS spectra of R6G. Comparison of R6G SERS spectra from this research (red line, bottom) and other reference (black line, top). The peak is borrowed from ref. 34. Typical SERS peaks of R6G are identical.

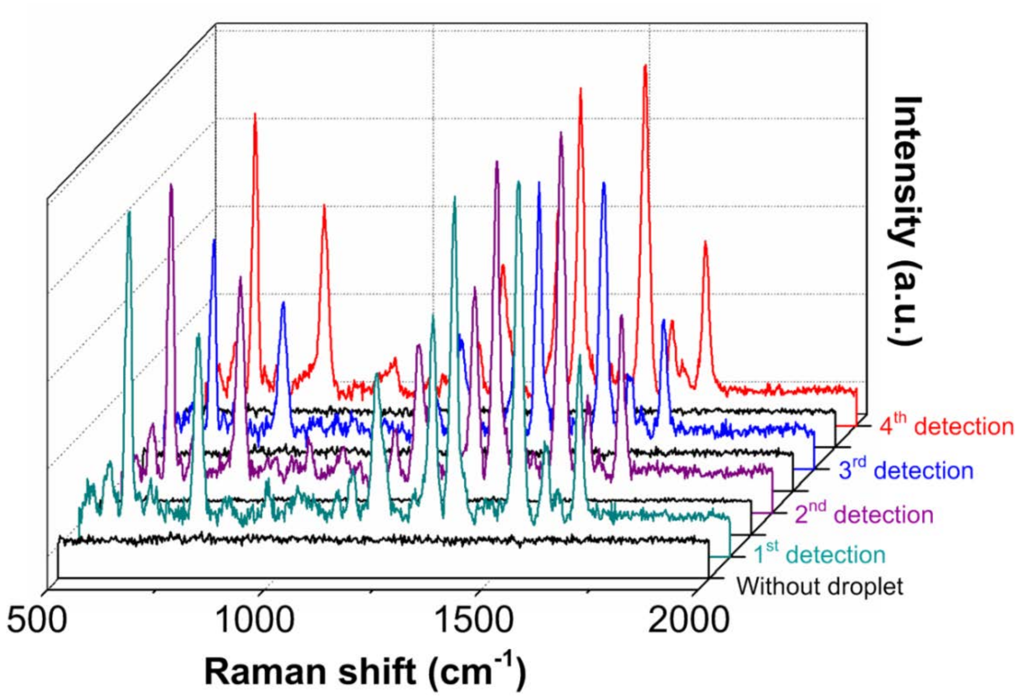

Supplementary Figure 8. Repetitive *in-situ* SERS measurements. Reproducibility of *in-situ* SERS measurements on the PDMS with micro-pillar arrays (10^{-3} M R6G).
